# Data on three-year flowering intensity monitoring in an apple orchard: A collection of RGB images acquired from unmanned aerial vehicles

**DOI:** 10.1016/j.dib.2023.109356

**Published:** 2023-07-05

**Authors:** Chenglong Zhang, João Valente, Wensheng Wang, Pieter van Dalfsen, Peter Frans de Jong, Bert Rijk, Lammert Kooistra

**Affiliations:** a Laboratory of Geo-information Science and Remote Sensing, Wageningen University & Research, Droevendaalsesteeg 3, 6708 PB Wageningen, the Netherlands; b Information Technology Group, Wageningen University & Research, Hollandseweg 1, 6706 KN Wageningen, the Netherlands; c Field Crops, Wageningen University & Research, Lingewal 1, 6668 LA Randwijk, the Netherlands; d Agricultural Information Institute, Chinese Academy of Agriculture Science, Beijing 100086, China; e Aurea Imaging BV, Nijverheidsweg 16B, 3534AM Utrecht, the Netherlands

**Keywords:** UAV, Flower blossom, Flower cluster, Yield mapping, Photogrammetry

## Abstract

There is a growing body of literature that recognises the importance of UAVs in precision agriculture tasks. Currently, flowering thinning tasks in orchard management rely on the decisions derived from time-consuming manual flower cluster counting in the field by an agrotechnician. Yet it is hard to guarantee the counting accuracy due to numerous human factors. The present dataset contains UAV images during the full blooming period of an apple orchard for three consecutive years, 2018, 2019, and 2020. It is directly linked to a research article entitled “Feasibility assessment of tree-level flower intensity quantification from UAV RGB imagery: A triennial study in an apple orchard”. The data collection site was an apple orchard located at Randwijk, Overbetuwe, The Netherlands (51.938, 5.7068 in WGS84 UTM 31U). Moreover, the flower cluster number and floridity ground truth are also provided in one row from the orchard. The UAV flights were conducted with different flying altitudes, camera resolutions, and lighting conditions. This dataset aims to support researchers focussing on remote sensing, machine vision, deep learning, and image classification, and the stakeholders interested in precision horticulture and orchard management. It can be used for flowering intensity estimation and prediction, and spatial and temporal flowering variability mapping by using digital photogrammetry and 3D reconstruction.

**Specifications Table**

**Table:** 

Subject	Computer Science and Pattern Recognition
Specific subject area	Remote Sensing, Machine vision, Deep learning, Image classification, Flower detection, Precision agriculture, Yield estimation
Data format	Raw
Type of data	Table, Image, Text
Data collection	The data are divided into three sets according to the collection years, 2018, 2019 and 2020. Different flying altitudes were conducted for each year. All the three field campaigns were taken during the full blooming period, in late April. The weather condition of data 2018 is overcast, and the other two years are sunny, which benefits the algorithm robustness development. Flying missions were set to autonomous mode, and images collected were geotagged.
Data source location	Institution: Field Crops, Wageningen University & Research City/Town/Region: Randwijk, Overbetuwe Country: the Netherlands GPS coordinates for collected data: WGS84 / UTM zone 31N (EPSG::32631)
Data accessibility	Repository name: Zenodo Data identification number: https://doi.org/10.5281/zenodo.6802308 Direct URL to data: https://zenodo.org/record/6802308#.YvvMFuxBz0p Instructions for accessing these data: All the data are compressed into a .zip file which can be downloaded directly via the link shared above. The data are available after decompressing.
Related research article	C. Zhang, J. Valente, W. Wang, L. Guo, A. Tubau Comas, P. van Dalfsen, B. Rijk, L. Kooistra, Feasibility assessment of tree-level flower intensity quantification from UAV RGB imagery: A triennial study in an apple orchard, ISPRS Journal of Photogrammetry and Remote Sensing 197 (2023) 256-273. https://doi.org/10.1016/j.isprsjprs.2023.02.003

## Value of the Data

• Precise flowering intensity estimation is the critical property of instructing flower thinning in orchards. This dataset presents visible aerial images of apple flowers and corresponding ground truth, flower cluster number, and floridity, during the full blooming period for three consecutive years. To our knowledge, this is the first time that a complete flowering dataset of stone fruit is publicly available, which is aimed at those interested in flowering estimation, yield estimation, and spatial and temporal flowering status mapping either based on 2D or on 3D data. It benefits the precision agriculture community and the growers with practical applications.
• The data is a contribution to the scientific communities of agriculture, machine vision, remote sensing, and robotics. More specifically, it advances the research focuses on precision horticulture and orchard management with the supply of publicly available datasets and ground truth.
• High-resolution orthomosaics and 3D coloured point clouds could be derived from the geotagged RGB images provided, by applying photogrammetry and 3D reconstruction. Thus, with these benchmark examples, researchers can develop and test new algorithms, such as advanced machine learning and deep learning, for object detection and classification, flowering intensity estimation and prediction, yield estimation, and spatial flowering variability mapping [1].
• The data provides multiple variations that reflect the challenges in apple or other stone fruit flowering intensity estimation and quantification. Datasets with different flying altitudes, image resolutions, and lighting intensity enable the evaluation of the impact of different parameters and the development of robust and generalized algorithms and models.
• One row in the apple orchard was prepared with a special setup for image acquisition by using easily distinguishable yellow observation tapes. The tapes divided individual trees into three sub-volumes, top, middle and bottom volumes, and the corresponding ground truth of these sub-volumes was also quantified in the field by an agrotechnician. This opens the possibility of inspecting flower distribution within each individual tree in detail.

## 1 Data Description

**Fig. 1:**
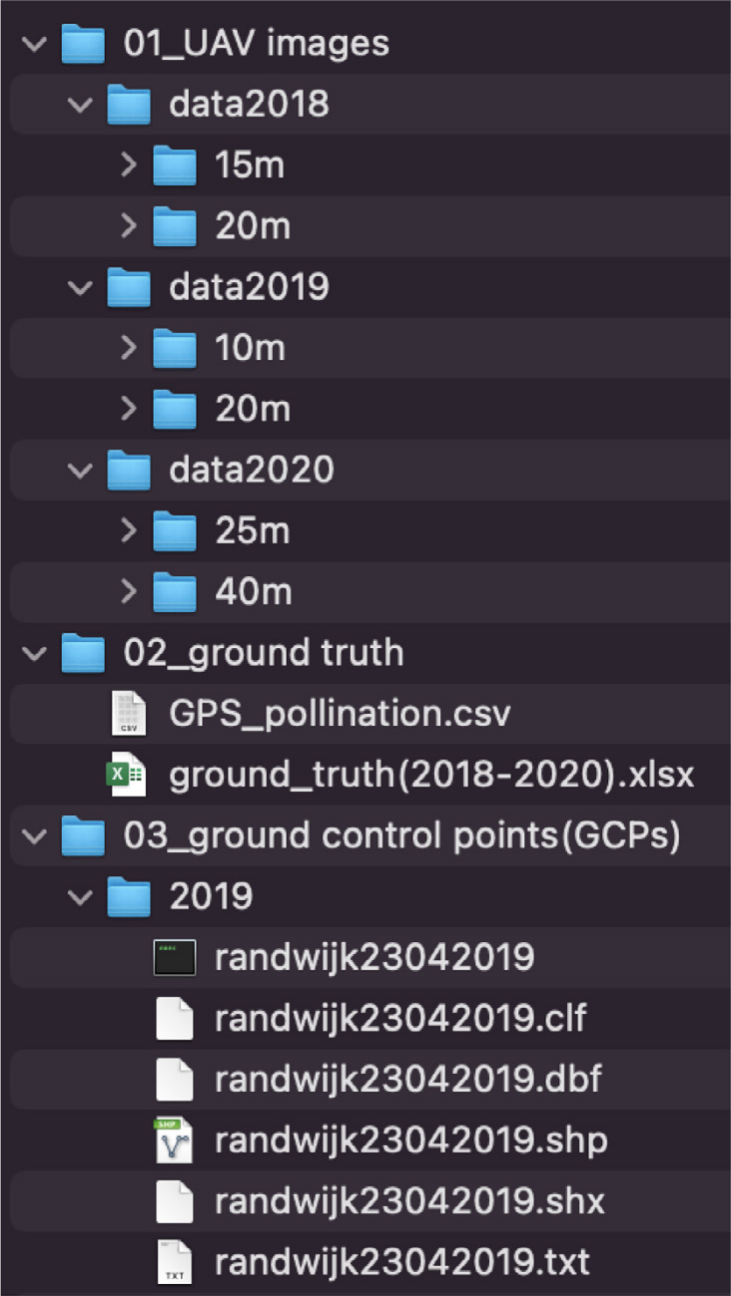
Overview of the flowering intensity dataset.

**Fig. 2:**
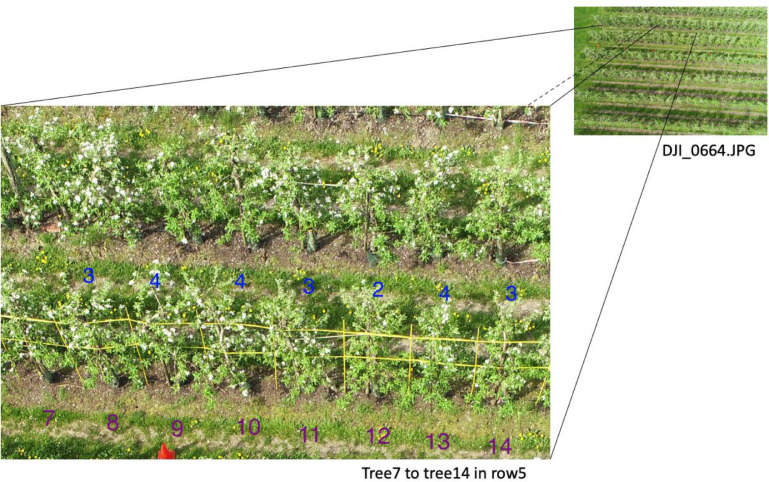
Samples of floridity from the UAV image in 2019. The purple number stands for the tree number while the blue number stands for the floridity score given by the expert.

**Fig. 3:**
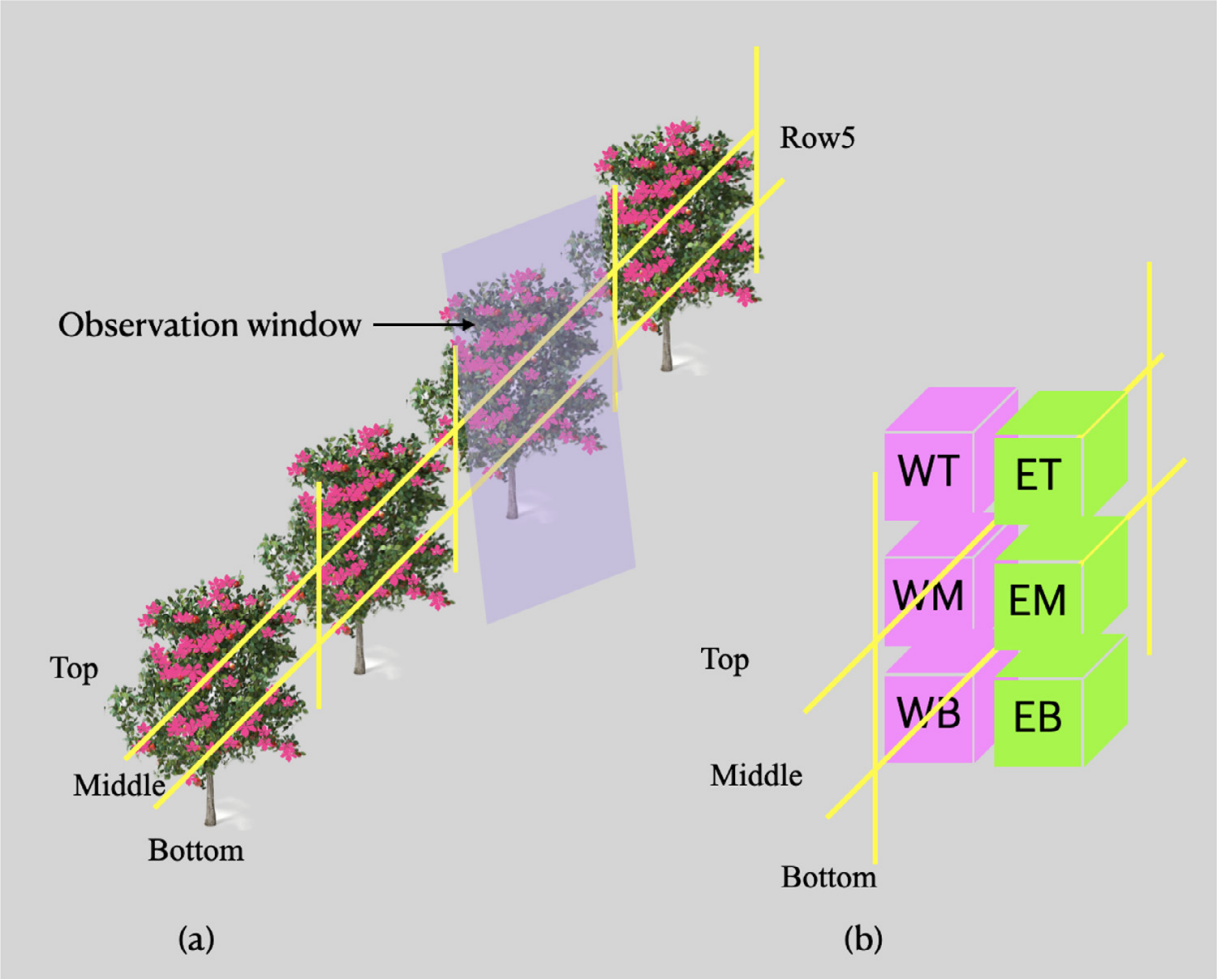
Environmental settings in row 5. WT: West top volume of a tree, WM: West middle, WB: West bottom, ET: East top, EM: East middle, EB: East bottom. Note: present figure was adapted from Fig. 2 in the related research article [2].

**Fig. 4:**
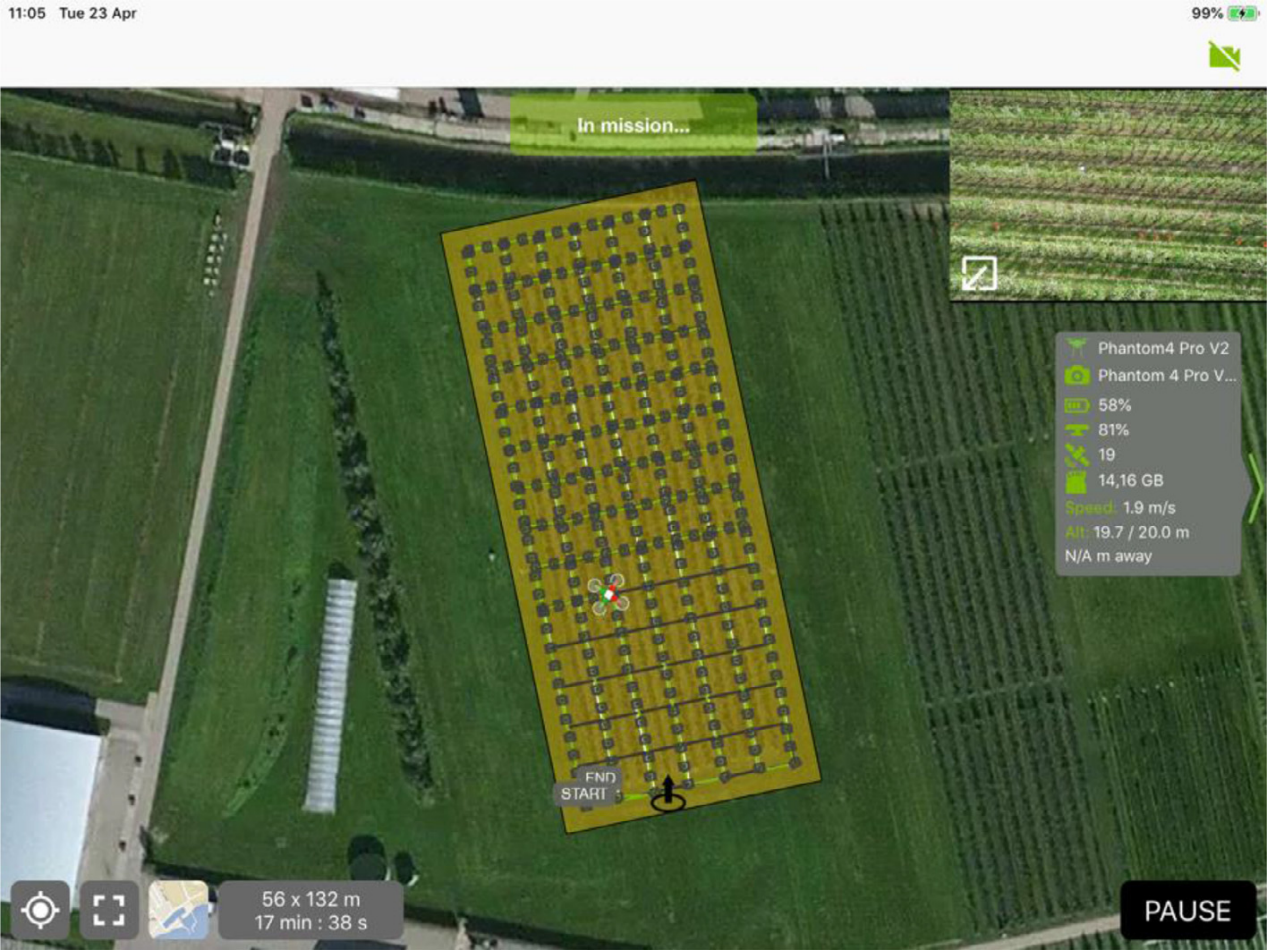
Screenshot from the remote control during a flight in 2019.

**Fig. 5:**
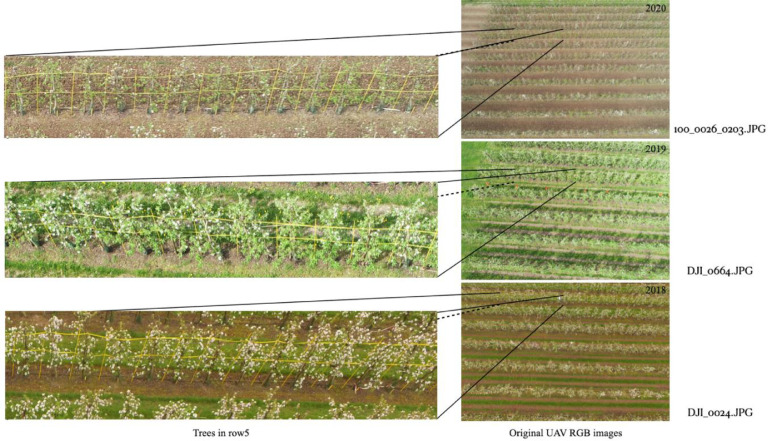
Samples of the three-year UAV images and the enlarged row 5 sections. Note: the present figure was adapted from Fig. 3 in the related research article [2].

The dataset describes UAV RGB images collected during the full blooming period of an apple orchard for three consecutive years, 2018, 2019 and 2020. Different UAV platforms and flying altitudes were applied, and various weather conditions were also covered. Detailed information was introduced in Table 1. Fig. 1 gives an overview of which types of data were collected. Fig. 2 and Fig. 5 provide samples of the UAV images collected. Fig. 2 also shows the trees with various floridity values given by an agronomist. Fig. 3 demonstrates the observation windows and the subvolumes of trees used by the agronomist for ground truth collection. Fig. 4 provides an example of the flying routine for automatic mapping missions.

**Table 1:** Statistical description of the generated dataset.

	Data 2018		Data 2019		Data 2020	
UAV platform	DJI Phantom 3 PRO, Shenzhen, China		DJI Phantom 4 PRO, Shenzhen, China		DJI Phantom 4 RTK, Shenzhen, China	
Sensor	FC300X		FC6310S		FC6310R	
Type	CMOS		CMOS		CMOS	
Resolution	4000 × 3000		5472 × 3648		5472 × 3648	
Focal length (mm)	3.61		8.8		8.8	
F number	f/2.8		f/4.5		f/2.8	
Exposure time	1/100		1/200		1/500	
Overlap ratio	85%		85%		85%	
Flying velocity (m/s)	2		1.9		2	
Flying altitude (m)	15	20	10	20	25	40
Data size	296	60	1002	418	252	120
Collection date & time	24^th^ April, 11:30 am		23^rd^ April, 10:53 am		18^th^ April, 11:33 am	
Weather	Overcast		Sunny		Sunny	
Temperature (°)	11		17		13	
Wind (km/h)	20 SW		20 E		15 NE	

*Note*: The present table was adapted from Table 1 as introduced in the related research article [2].

When the compressed file uploaded to the repository of Zenodo is decompressed, the dataset is organized in three folders: UAV images, ground truth, and ground control points (GCPs) (Fig. 1).

The UAV images folder includes three sub-folders, data2018, data2019, and 2020, storing the original UAV RGB images collected in the year 2018, 2019, and 2020, respectively. In each folder, sub-folders were created according to the flying height applied. For example, in the folder data2019, sub-folder 10m and 20m store 1002 and 418 UAV images collected with the flying height of 10m and 20m, respectively. In addition, detailed data collection materials, settings, and weather condition information were also assessed (Table 1). It should be noticed that, in 2020, half of the trees in the orchard were removed.

The ground truth was collected up to two days before or after the UAV data collection. The ground truth consists of two types: 1) Flower cluster number and 2) floridity index per tree. The ground truth was assessed for three years and saved in the .xlsx file named ground_truth (2018-2020) (Fig. 1). A flower cluster is a group of mostly five flowers that develop from one bud. Generally, one flower cluster contains three to six flowers. Flower cluster data was counted manually for one tree row only, row 5, and it stands for the exact number of clusters on each individual tree. Floridity was an index assessed by an agrotechnician in the field giving each tree a flowering intensity rate ranging from 1 to 9. Here a score of 1 represents no flowers and 9 is heavily blooming. More specifically, the expert in the orchard firstly scores every tree in this row from the West and East side. The average floridity, as the mean of the East and West side score, is regarded as the floridity for the tree. Fig. 2 shows the samples of floridity assessment results from the East side of row5. The ideal situation in fruit growing is a floridity of 5 (intermediate bloom).

In the file ground_truth (2018-2020) stored in the ground truth folder, detailed flower clusters and floridity were recorded in 9 sheets. The first sheet in the .xlsx file shows the GPS position of the trees in row5 where latitude and longitude data was measured for each tree. Based on the unique environmental settings in row 5 (Fig. 3), the cluster number in the six sub-volumes, the total cluster number in the tree, and in the observation window were counted for the three years. This detailed counting was applied for 32, 31, and 19 trees in row 5, in 2018, 2019, and 2020, respectively. Floridity data was measured for all the trees in row 5 for the three years. Moreover, two additional rows, row 2 and row 7, were also assessed for 2019 and 2020. The other file, the .csv file, in the ground truth folder records the GPS position of the pollination trees. These pollinators were planted between the apple trees to promote pollination. In total 144 pollinator trees were planted for this experimental purpose and the latitude and longitude of each tree were measured. When the flower clusters and floridity were measured, these pollinator trees were excluded.

The ground control points (GCPs) folder includes the files that record the coordinates of several points on the ground for the year 2019 aiming at improving the mosaicking and 3D reconstruction [3]. The same data was stored in five file formats: .clf, .dbf, .shp, .shx, and .txt for different data read requirements. In 2019, six GCPs were used for the whole orchard. Two GCPs were set in the middle part of the orchard and the other four points were measured at the four corners. In the case of 2020, the UAV platform used was DJI Phantom 4 RTK (Table 1). Then no GCPs were used.

## 2 Experimental Design, Materials and Methods

The data collection site was an 0.47ha apple orchard located at Randwijk, Overbetuwe, The Netherlands (51.938, 5.7068 in WGS84 UTM 31U). The cultivar is Elstar, *Malus pumila ‘Elstar’*, and M9 is the rootstock. There were 14 rows and in each row around 101 trees were planted. Row spacing and tree spacing are 3.0m and 1.1m, respectively. At the beginning of 2020, half of the trees were removed for orchard management. More specifically, tree numbers ranging from 1 to 45 were removed for each row in the orchard. Row 5 was prepared with a special setup for flower cluster counting (Fig. 3a), which also benefits the research on intra-tree flower intensity monitoring. Easily identifiable yellow tapes and poles were used to delineate each individual tree and divide each tree into six sub-volumes (Fig. 3b). Yellow poles were vertically sticked in the middle of two adjacent trees in row 5 while yellow tapes were placed horizontally aiming at dividing the trees into three equal sub-volumes, top, middle, and bottom. Based on this setting, each tree in row 5 situates between two yellow poles, and the space between these two poles is regarded as the observation window for detailed flower cluster counting.

Data on apple flowering intensity was collected for three continuous years, 2018, 2019, and 2020. Every year a different UAV with a specific RGB sensor was adopted (Table 1). These three UAVs use quick-release propellers. In 2019, handheld real-time kinematic (RTK) was also used to measure GCPs. While Phantom 4 RTK adds an RTK positioning module and a TimeSync system which enable data acquisition without additional GCPs measurement. The data acquisition date was selected for the time that the apple trees were at the full blooming stage and that suitable weather conditions for flights (not rainy) were available (Table 1). This date changed every year with climatic and phenological changes. At the same time, two types of ground truth, flower cluster, and floridity were manually counted and assessed by the experts in the local orchard. Data collection under various flying heights was designed for three years in order to achieve statistical study [4]. All the flying missions were set to automatic flying models with DJI apps, such as DJI GO (Fig. 4). The resulting UAV images are shown in Fig. 5.

## 3 Limitations

Not applicable.

## Ethics Statement

The authors declare that the present dataset meets the ethical requirements of Data in Brief and that no animal or human study was involved.

## CRediT Author Statement

**Chenglong Zhang**: experiment design, data collection, writing manuscript draft; **João Valente**: data collection, supervision, manuscript editing; **Wensheng Wang**: supervision, manuscript editing; **Pieter van Dalfsen** & **Peter Frans de Jong**: ground truth collection; **Bert Rijk**: data collection; **Lammert Kooistra**: experiment design, data collection, supervision, manuscript editing.

## Declaration of Competing Interests

The authors declare that they have no known competing financial interests or personal relationships that could have appeared to influence the work reported in this paper.

## Data Availability

Data on three-year flowering intensity monitoring in an apple orchard (Original data) (Zenodo).

## References

[1] Zhang C., Valente J., Kooistra L., Guo L., Wang W. (2021). Orchard management with small unmanned aerial vehicles: a survey of sensing and analysis approaches. Precis. Agric..

[2] Zhang C., Valente J., Wang W., Guo L., Tubau Comas A., van Dalfsen P., Rijk B., Kooistra L. (2023). Feasibility assessment of tree-level flower intensity quantification from UAV RGB imagery: a triennial study in an apple orchard. ISPRS J. Photogramm. Remote Sens..

[3] Torres-Sanchez J., Lopez-Granados F., Serrano N., Arquero O., Pena J.M. (2015). High-throughput 3-d monitoring of agricultural-tree plantations with Unmanned Aerial Vehicle (UAV) technology. PLoS One.

[4] Zhang C., Valente J., Kooistra L., Guo L., Wang W. (2019). Opportunities of UAVs in orchard management. Int. Arch. Photogramm. Remote Sens. Spatial Inf. Sci..

